# The Relationship Between Mesenchymal Stem Cells and Tumor Dormancy

**DOI:** 10.3389/fcell.2021.731393

**Published:** 2021-10-12

**Authors:** Linxian Zhao, Kai Zhang, Hongyu He, Yongping Yang, Wei Li, Tongjun Liu, Jiannan Li

**Affiliations:** ^1^Department of General Surgery, The Second Hospital of Jilin University, Changchun, China; ^2^Operating Theater and Department of Anesthesiology, The Second Hospital of Jilin University, Changchun, China

**Keywords:** mesenchymal stem cells (MSCs), cancer stem cells (CSCs), disseminated tumor cells (DTCs), tumor dormancy, anti-tumor treatment

## Abstract

Tumor dormancy, a state of tumor, is clinically undetectable and the outgrowth of dormant tumor cells into overt metastases is responsible for cancer-associated deaths. However, the dormancy-related molecular mechanism has not been clearly described. Some researchers have proposed that cancer stem cells (CSCs) and disseminated tumor cells (DTCs) can be seen as progenitor cells of tumor dormancy, both of which can remain dormant in a non-permissive soil/niche. Nowadays, research interest in the cancer biology field is skyrocketing as mesenchymal stem cells (MSCs) are capable of regulating tumor dormancy, which will provide a unique therapeutic window to cure cancer. Although the influence of MSCs on tumor dormancy has been investigated in previous studies, there is no thorough review on the relationship between MSCs and tumor dormancy. In this paper, the root of tumor dormancy is analyzed and dormancy-related molecular mechanisms are summarized. With an emphasis on the role of the MSCs during tumor dormancy, new therapeutic strategies to prevent metastatic disease are proposed, whose clinical application potentials are discussed, and some challenges and prospects of the studies of tumor dormancy are also described.

## Introduction

Although clinical treatments are developing, cancer is still a significant challenge due to recurrence and metastasis. The unbridled proliferation and uncontrollable dormancy have been regarded as two main hallmarks of cancer (Yeh and Ramaswamy, 2015). Recent studies have discovered a direct association between cancer progression and tumor dormancy (Boire et al., 2019). In other words, tumor dormancy is one of the leading causes of tumor outgrowth, relapse, and metastasis. In 1934, tumor dormancy was firstly defined by Willis as an intricate phenomenon that tumor cells stop proliferating but remain capable of malignant progression (Willis, 1953). In recent years, tumor dormancy has been extensively investigated. Tumor dormancy can occur in various phases of tumor progression, which is a primary cause of tumor recurrence even the primary tumor has been resected for decades (Kleffel and Schatton, 2013). Therefore, a deep understanding of the mechanisms of tumor dormancy is needed to develop more promising therapeutic strategies to eliminate dormant cells.

Based on the physiological traits, tumor dormancy can be divided into two categories: cellular dormancy and tumor mass dormancy. Cellular dormancy, a quiescence of solitary cells, is characterized by each cancer cell residing in the G0-G1 phase of the cell cycle. Besides, the underlying mechanisms of cellular dormancy, governed by the extracellular matrix, nutriture, and metastatic microenvironment, are complicated (Dittmer, 2017; Endo and Inoue, 2019). Tumor mass dormancy is characterized by entire neoplasm cells entering a balance between cell division and apoptosis, mediated by angiogenic dormancy and immunologic dormancy (Gao et al., 2017; Hen and Barkan, 2020). With the changes in the surrounding environment, these cessative cells can re-enter the cell cycle, leading to cancer recurrence. Likewise, the progression of cancer occurs in tumor mass dormancy when angiogenic dormancy and immune dormancy are reawakened by surrounding stimuli, after which the balance between proliferation and apoptosis will be broken (Yeh and Ramaswamy, 2015), leading to cancer recurrence. Besides, tumor dormancy is not a single state but an equilibrium determined by cell-intrinsic characteristics and surrounding niche (Senft and Jeremias, 2019).

Stem cells are units of biological organization with clonogenic potential and self-renewal capacity and can be differentiated into multiple functional cell lineages (Dulak et al., 2015). Up to now, stem cells have not been clearly defined due to elusive identities. Therefore, the classifications of stem cells are varied. There are two main classification criteria. In terms of development potential, stem cells can be divided into totipotent stem cells, pluripotent stem cells, and pluripotent stem cells. Totipotent stem cells are capable of differentiating into various kinds of cells and developing into complete human beings (Zakrzewski et al., 2019). Pluripotent stem cells are accompanied with the potential of differentiating into multi-tissue but don’t possess the ability to develop into a complete individual (Abu-Dawud et al., 2018). Unipotent stem cells, derived from further differentiation of pluripotent stem cells, can only differentiate into one or two closely related types of cells (Dulak et al., 2015).

On the other hand, it has been proposed that the main sources of stem cells include embryonic tissues, adult tissues, fetal tissues, and differentiated cells with genetically reprogrammed (Qin et al., 2016). Therefore, based on their origins, stem cells can be divided into four categories: embryonic stem cells, fetal stem cells, adult stem cells, and cord blood stem cells (Li et al., 2020). Embryonic stem cells possess the capability of totipotent stem cells, which have a good potential for differentiation (Dou et al., 2020); Fetal stem cells have a higher potency of multi-differentiation than adult stem cells and can differentiate into numerous cell phenotypes (Bacakova et al., 2018). Adult stem cells are multipotent and are capable of differentiation into only a limited number of cell types, and therefore can only be used to repair and regenerate elected tissue (Han et al., 2019). Cord blood stem cells include hematopoietic stem cells (HSCs) and MSCs (Mohr and Zwacka, 2018). HSCs are generally isolated from bone marrow and have been successfully used in clinical treatment for 50 years, especially in hematological malignancies (Bujko et al., 2019). In recent years, MSCs also have shown great application potential and are mainly applied to treat tumors and various systemic diseases (Pajarinen et al., 2019). Details about MSCs are summarized in Chapter 4.

The research interest in stem cells is due to the potential application in a wide range of clinical treatments, including tissue engineering, cell replacement therapy, gene therapy, system reconstruction, and anti-aging treatment. However, the underlying mechanisms have not been thoroughly studied, which also promotes more researchers to focus on these unknown fields. In recent years, an increasing number of studies have been performed to investigate the interaction between stem cells and tumor development. Especially, with the discovery of cancer stem cells, the roots of uncurable cancer are gradually being ascertained. Among all stem cells, MSCs have been one of the most promising ones in cancer treatment. This review summarizes the mechanisms of MSC-mediated modulation of tumor dormancy and discusses the relationship between cancer stem cells and dormant tumor cells.

## Cancer Stem Cells and Disseminated Tumor Cells

In 1997, Bonnet et al. first isolated CSCs from patients of acute myeloid leukemia (Bonnet and Dick, 1997), and discovered that these CSCs show similar characteristics with normal stem cells, which are heterogeneous and also possess the capacity of self-renewal and multi-differentiation (Desai et al., 2019). During the next decades, it has been further confirmed that CSCs are a small subset of cancer cells that are resistant to conventional therapies and can initiate tumor formation (Eltoukhy et al., 2018b). Nowadays, it has been reported that the mechanisms of CSCs formation can be explained in two ways, namely the gene mutations of normal stem cells and the dedifferentiation of progenitor cells (Scheel and Weinberg, 2012; Crea et al., 2015; Eltoukhy et al., 2018b), also showing that CSCs are not a single subpopulation but a group of multi-source cells.

Based on the differentiation capacity, CSCs can be divided into dormancy-competent CSCs (DCCs) and dormancy-incompetent CSCs (DICs) (Talukdar et al., 2019). In this review, DCCs are mainly discussed as being capable of escaping immune clearance and entering dormancy and being responsible for tumor progression, metastasis, chemoresistance, as well as recurrence (Jones et al., 2012; De Angelis et al., 2019). Further studies have discovered that there are many similar characteristics and signaling pathways between DCCs and dormant tumor cells (Talukdar et al., 2019). For instance, it has been reported that DCCs and dormant cancer populations both can maintain quiescence, delay cancer recurrence, resist tumor therapy, and evade immunologic clearance (Kleffel and Schatton, 2013), indicating that there may be a direct relationship between DCCs and tumor dormancy. In other words, DCCs may be the main subtype of dormant tumor cells. Interestingly, besides DCCs-derived dormant tumor cells, non-DCCs-derived ones have also been discovered in many cancers, also suggesting that non-CSC subpopulations also have the capacity of becoming quiescent (Talukdar et al., 2019).

Disseminated tumor cells (DTCs) were first discovered in the bone marrow. These cells not only play a pivotal role in the growth, dormancy, early metastasis, and late recurrences of minimal residual disease (Goddard et al., 2018), but also mark the early stage of cancers and have been used to monitor tumor progression (Synnestvedt et al., 2012). To date, the specific source of DTCs is still unclear. However, recent studies proposed that DTCs could be derived from circulating tumor cells (CTCs) and CSCs at all stages of tumor progression (Crea et al., 2015; De Angelis et al., 2019). In addition, by analyzing cell phenotype and function, researchers have discovered that DTCs and CSCs both possess similar dormant stem-cell-like abilities, such as immune evasion, chemoresistance, and the ability to increase the likelihood of recurrence (Hen and Barkan, 2020). By definition, DCCs and DTCs, the progenitor cells of tumor dormancy, both have the potential ability to become dormant tumor cells (Jahanban-Esfahlan et al., 2019; Talukdar et al., 2019). For example, the latest research has found that the CSCs of breast cancer could lurk in the bloodstream and premetastatic sites as CTCs and DTCs during the early stage of the primary tumor for two decades (De Angelis et al., 2019).

Moreover, DTCs are involved in the formation of tumor dormancy mediated by the organ microenvironment and immune system (Sosa et al., 2014; Risson et al., 2020). For instance, the dormant DTCs can evade immune system recognition and seed in bone marrow or distant tissues as the root of future metastasis (Hosseini et al., 2016; Risson et al., 2020), suggesting that tumor metastases may occur during the early phases of tumor progression. In conclusion, these findings indicate that DTCs and DCCs, like many sleeping seeds, are undetectable but can switch between the state of sleeping and proliferation (Hen and Barkan, 2020). Besides, the cell intrinsic or microenvironmental factors can also determine whether a niche is permissive or not. The bone marrow, a kind of particularly permissive microenvironment for tumor cells, can maintain DCCs in a dormant state for many decades before reactivation and ultimate metastatic outgrowth (Hen and Barkan, 2020). To prevent cancer metastasis, in future studies, what initiates CTCs/DTC quiescence and what promotes them to re-enter the cell cycle need to be clarified.

## The Mechanisms of Tumor Dormancy

Up to now, the mechanisms of tumor dormancy have only been partly illuminated, which can be divided into two categories: intracellular factors such as genetic alterations and the changes of signaling pathway; and extracellular factors such as tumor microenvironments, acidosis, hypoxia, angiogenic switch, as well as immunologic dormancy (Triana-Martinez et al., 2020). Therefore, only by deciphering mechanisms underlying MSC-mediated regulation of tumor dormancy, can more efficient targeted therapies be developed and the safe clinical application of MSCs be ensured.

### Intracellular Mechanisms

#### Genetic Alterations

Genetic alteration not only contributes to tumorigenesis but also induces dormancy of cancer cells. It has been proposed that the mechanisms of DCCs entering dormancy are reversible genetic alterations due to microenvironmental stress such as hypoxic, lack of nutrition, and exogenous conditions. However, these alterations must be controlled within a reasonable range. If overaccumulation of mutations happens, DCCs will lose the dormancy potential and gradually become inadaptable to the bone marrow niche, leading to unrestrained tumor growth and multi-therapy-resistant cancers (Husemann et al., 2008; Crea et al., 2015). Furthermore, recent studies reported that some genetic alterations correlated with cell proliferation and/or differentiation which are involved in initiating and maintaining the dormant phenotype. For example, the upregulated KiSS1 gene can maintain the dormant state of melanoma, ovarian cancer, and breast cancer in preclinical cancer models (Gomatou et al., 2021).

#### Intracellular Signaling Mechanisms

As another key player of tumor dormancy, Mitogen-activated protein kinase (MAPK) pathways can instruct cells to respond to extracellular stimuli. MAPK family is a paramount mediator during cellular processes such as cell growth, differentiation, migration, proliferation, survival, and innate immunity (Johnson et al., 2005; Krens et al., 2006). Based on the composition of the signal transduction pathway, the MAPK family can be divided into three signaling cascades subfamilies: the extracellular signal-regulated kinases (ERK MAPK, Ras/Raf1/MEK/ERK), which is one of the most essential cascades for cell proliferation (Fang and Richardson, 2005), and is mainly activated by growth factors (Garrington and Johnson, 1999); the c-Jun amino-terminal kinases, which mainly regulates cell growth and cell apoptosis (Ip and Davis, 1998), and is activated by stress factors, differentiation factors, and growth factors (Weston and Davis, 2007); the p38 MAP kinases, which consist of p38α, p38β, p38γ and p38, all of which can be activated by stress factors and are essential for cell proliferation and differentiation (Nebreda and Porras, 2000; Krens et al., 2006). In terms of tumor dormancy, the effect of the different signaling pathways of the MAPK family is different. For instance, the activated p38 MAPK signaling pathway can induce DTCs into a quiescent status (Sosa et al., 2011) while the activated Jun N-terminal kinase pathway can promote tumor proliferation and outgrowth (Sui et al., 2014). Besides, Jo et al. have proposed that tumor cells lacking nutrients could enter into quiescence via reducing PI3K-AKT signaling (Jo et al., 2008). In addition, the extracellular signals also regulate tumor dormancy, such as the transforming growth factor-β (TGF-β) family and bone morphogenetic proteins (BMPs), which will be discussed in the rest of this paper.

### Extracellular Mechanisms

#### Tumor Microenvironments

In comparison, extracellular factors are more complicated. It has been suggested that tumor dormancy is mainly regulated by tumor microenvironments, a complex network including extracellular matrix, bone marrow stem cell niche, perivascular niche, CSC niche, and so forth (Gay and Malanchi, 2017; Talukdar et al., 2019). Figuratively speaking, the tumor microenvironments are similar to the ‘soil’ in the ‘seed and soil’ theory, which was first proposed by James Paget at the end of the 19th Century (Phan and Croucher, 2020), and only when the ‘soil’ is fertile and suitable for the tumor cell (the ‘seed’), will the dormant tumor cells start to re-proliferate. How these niches induce and maintain tumor dormancy and promote cell cycle arrest is not fully understood. The hypothesis is proposed that the bone marrow may provide a pro-dormancy signal to tumor cells. For example, it has been reported that most of the DTCs residing in the tumor niche are successfully eradicated by anti-tumor treatments and a few of them resided in bone marrow could enter into the state of quiescence and be mediated by bone marrow niches (Widner et al., 2018). Noteworthy, the microenvironmental factors may be one of the most complicated mechanisms of tumor dormancy, including many biochemical and biophysical factors, which can influence tumor dormancy by affecting interactions among myriad molecular (Talukdar et al., 2019). In conclusion, tumor dormancy is regulated by a series of complex cues, which are still unclear due to contextual molecular mechanisms and various undiscovered factors despite a series of plausible theories and hypotheses have been proposed. It has shown that tumor cells possess a mass of mutated signaling pathways to interact with cytokines secreted by various cells or tumor microenvironment.

#### Acidosis

The acidity of the extracellular microenvironment is the main characteristic of solid tumors, which is also correlated with the poor prognosis of many malignancies (Peppicelli et al., 2017). In recent years, more studies have focused on the relationships between the extracellular acidosis microenvironment and tumor dormancy. The underlying mechanisms of how extracellular acidosis may contribute to tumor cell dormancy has also been discussed. Current studies have reported that acidic tumor microenvironment was capable of promoting tumor dormancy through various mechanisms, such as increasing the percentage of cells in the G0 phase, regulating growth factor signaling and Raf/ERK pathway, increasing phosphorylation metabolism, leading to high resistance to apoptosis and autophagy, as well as hiding from immune surveillance (Peppicelli et al., 2017). In addition, the sustained extracellular acidity can reduce the intracellular pH from neutral to below pH 6.5, thus directly affecting both tumor cell and normal cell cycle through downregulating cyclin-dependent kinase 1-cyclin B1 activity (Putney and Barber, 2003). Interestingly, acidity can also awake the dormant tumor cells with genetic or epigenetic changes, leading to tumor recurrence, local invasion, and metastasis (Peppicelli et al., 2017). In MSCs, another study found that MSCs can be induced to secrete high levels of TGFβ family members in an acidic microenvironment, thus contributing to tumor dormancy (Peppicelli et al., 2015). Moreover, a low pH can facilitate tumor-recruited MSCs to secrete pro-tumorigenic cytokines. For example, in osteosarcoma, acidosis can lead to the reprogramming of tumor-recruited MSCs to the secretion of osteosarcoma-supporting mediators, such as IL6, IL8, and NF-κB inflammatory pathway (Di Pompo et al., 2021).

#### Hypoxia

A hypoxic microenvironment is very common in various malignancies, especially in solid tumors caused by rapid tumor cell growth and disorganized angiogenesis.

Previous study has reported that hypoxia could lead tumor cells to resist traditional treatment and induce a malignant tumor phenotype (Pouyssegur et al., 2006). In recent years, to better mimic hypoxic microenvironment and investigate the relationship between hypoxia and tumor dormancy, many *in vitro* dormancy models have been performed, such as hypoxic chamber, adding iron-binding/substitute agents, as well as imposing diffusion-limited hypoxia culturing cells in 3D hydrogel systems (Butturini et al., 2019). Further study proposed that the hypoxia-resistant breast cancer cell line could enter into a reversible dormant status in reoxygenation conditions, which might be caused by cells autophagy (Carcereri de Prati et al., 2017). A similar phenomenon was also observed in colorectal cancer and urothelial carcinoma (Endo et al., 2014). Other studies also have proposed that hypoxia was involved in regulating tumor dormancy by influencing the expression of molecules, such as angiostatin, thrombospondin, epoxyeicosatrienoic acids, as well as vascular endothelial growth factor (Gomatou et al., 2021).

#### Angiogenic Switch

In addition to the above mechanisms, the angiogenic switch has been proved to play a key role in regulating tumor dormancy (Shaked et al., 2014). With the progression of the tumor, the angiogenic capacity must be matched to the rate of tumor cell growth (Folkman, 1971). In other words, if tumor mass cannot acquire sufficient angiogenic potential during the tumor progression, these tumor cells will enter into a dormant status. The angiogenic dormancy can be reckoned as a stage of tumor progression when the pro- and anti-angiogenic roles achieve a balance. The angiogenic switch refers to the transition from the inability of angiogenesis to the acquisition of angiogenic potential, resulting in growing vascularized tumors (Hanahan and Folkman, 1996). It has been further confirmed that the angiogenic switch is regulated by angiogenic factors and oncogene expression (Ribatti et al., 2007; Natale and Bocci, 2018). These angiogenic factors are derived from tumor cells or other cells residing in the tumor microenvironment. The expression of these factors can be influenced by environmental stress and genetic changes, such as activation of oncogenes and inhibition of tumor suppressor genes (Kerbel, 2008). For example, it has been reported that hypoxia signaling and oxygen species are involved in the regulation of tumor dormancy by generating growth factors such as urokinase receptor, focal adhesion kinase, and epidermal growth factor receptor (Talukdar et al., 2019).

#### Immunologic Dormancy

Plenty of studies have reported that the immune system plays critical roles in regulating and maintaining tumor dormancy (Kleffel and Schatton, 2013; Hu et al., 2020). Current studies have found that T lymphocytes and CD8+ T cells are involved in regulating immune-mediated cancer dormancy at the metastatic site (Jahanban-Esfahlan et al., 2019). Particularly, an association of immune cells and their inflammatory mediators are involved in immunological dormancy. For instance, it has been proposed that the immune cells residing in the bone-tumor microenvironment could produce pro-inflammatory cytokines and anti-inflammatory cytokines, called the “bone-tumor-inflammation network” to mediate inflammatory responses (Hu et al., 2020). Another study found that DTCs were capable of escaping from immune-surveillance and resulted in tumor escape (Endo and Inoue, 2019). Accordingly, we can improve the efficacy of tumor immunotherapy by utilizing immunotherapy treatments to target disseminated cancer dormancy, aiming to control cancer growth and even eliminate tumors.

## Mesenchymal Stem Cells

Mesenchymal stem cells (MSCs) are a subset of stromal cells that possess the ability of multi-lineage differentiation and self-renewal (Ding et al., 2011). To be more specific, MSCs are reckoned as one subtype of adult stem cells, which not only have the capability of normal stem cells, but also show some unique multipotent properties (Kim and Kim, 2019) such as immune privilege, tumor homing feature, and anti-tumor ability (Eltoukhy et al., 2018b), making MSCs the most promising stem cells for curing cancer and ensuring safe clinical applications and trials. It has also been suggested that the effect of MSCs on tumor growth is contradictory, which can play anti-tumor or pro-tumor roles on tumor development (Bartosh et al., 2016). It can be concluded that MSCs are the most commonly used stromal cells in anticancer experimental or clinical researches because of their great therapeutic potential.

Current studies have shown that MSCs could interact with tumor cells and their niches through the paracrine pathway and physical mechanisms (Cammarota and Laukkanen, 2016), to mediate the growth of a primary tumor and the spread of distant metastasis (Ridge et al., 2017; Ahn, 2020). For example, the bone marrow-derived MSCs (BMSCs) can induce breast cancer cells into dormancy via the paracrine route to activate the JAK/STAT3 signaling pathway (Leyh et al., 2015), which partly illuminates the reasons why the metastatic latency period of breast cancer can prolong to 25 years after primary tumor resection. Studies have also found that whether MSCs promote or inhibit tumor progression and formation (Timaner et al., 2020) depends on the type of tumor, the source of MSCs, as well as tumor microenvironment. For example, relative studies have discovered that adipose tissue-derived MSCs (AMSCs) could promote the growth of brain tumors, ovarian tumors, gastric tumors, and breast tumor (Lim et al., 2016; Li et al., 2020), but inhibit the growth of melanoma both *in vitro* and *in vivo* (Ahn et al., 2015). Another study has proposed the allogeneic MSCs could induce melanoma cells into a malignant tumor (Djouad et al., 2003).

In 1986, Dvorak first proposed that the tumors are wounds that do not heal (Dvorak, 1986). In recent years, with the further research of cancer, it has been considered that MSCs are capable of regenerating and promoting wound healing (Lyu et al., 2020). However, tumor “wound” is different from conventional tissue wound, and MSCs can aggravate these non-healing wounds by directly or indirectly interacting with the tumor wound microenvironment (Li et al., 2019). For example, tumor-associated MSCs (TA-MSCs) usually promote conventional tissue wound healing via accelerating inflammation response and suppressing adaptive immunity. However, these two mechanisms are beneficial for tumor progression, resulting in cancer wound overhealing (Li et al., 2019). On the other hand, the bidirectional interaction between MSCs and tumor cells can result in constant stroma renewal, which is similar to a wound that never heals (Cammarota and Laukkanen, 2016).

In addition to the above studies, studies on the relationship between MSCs and tumor dormancy have also been extensively concluded in the antitumor field. During the migration into the tumor niche, MSCs can crosstalk with tumor cells and shape cancer phenotype (Spaeth et al., 2008). In this paper, the ways of different sources of MSCs influence tumor dormancy are summarized, providing more opportunities and challenges for researchers to focus on this field.

## The Influence of MSCs on Tumor Dormancy

**FIGURE 1:**
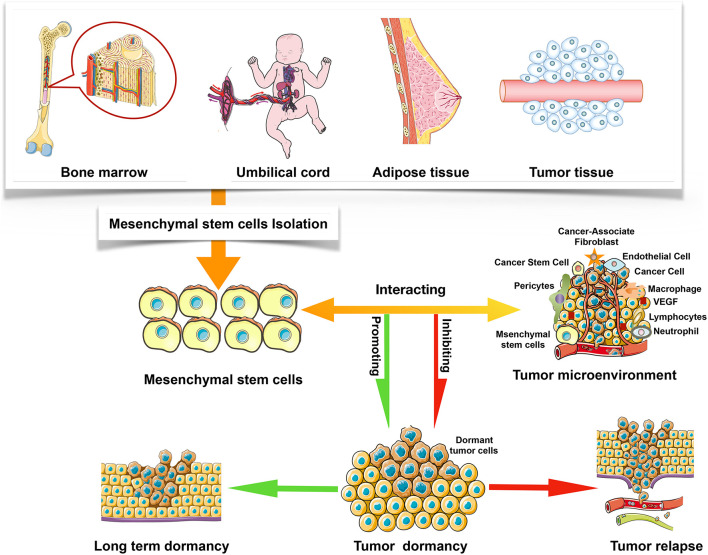
The sources of MSCs and the interaction between MSCs and tumor microenvironment.

Mesenchymal stem cells have been discovered in most organs, including the menses blood, endometrial polyps, bone marrow, fetal tissues, umbilical cord matrix, and adipose tissue (Ding et al., 2011; Murphy et al., 2013; Li et al., 2020), but current studies have shown that different sources of MSCs possess distinct characteristics. These differences also partly illustrate why MSCs with different sources can play even opposite effects on tumor progression. It has been discussed in this paper that MSCs could either inhibit or promote tumor growth, but the specific mechanism is still controversial (Galderisi et al., 2010). Notably, tumor microenvironment plays an important role in regulating tumor dormancy, including the inhibition and promotion effects (Figure 1). For instance, TA-MSCs and BMSCs have different phenotypes, which may be influenced by tumor microenvironment factors, such as cancer cytokines and secretory protein (Cammarota and Laukkanen, 2016). In this review, the effects of BMSCs, hUCMSCs, TA-MSCs, and AMSCs on tumor dormancy are summarized. Table 1 shows the effect of MSCs on tumor dormancy.

**TABLE 1:** The effect of MSCs on tumor dormancy.

**MSCs types**	**The effect on tumor dormancy**	**Factor/signal**	**Mechanism**	**Brief introduction**	**References**
BMSCs	Dual role	TGF-β1	(1). Promoting breast cancer cell proliferation by inhibiting the angiogenic dormancy (2). Inducing squamous cell carcinoma latency by regulating cell-cycle gene transcription to control a reversible G1 cell-cycle arrest.	In terms of different tumors, TGF-β1 can play distinct roles on tumor dormancy.	Ghajar et al., 2013; Brown et al., 2017; Jahanban-Esfahlan et al., 2019
	Promoting	TGF-β2 GAS6	(1). Driving CSCs and DTCs into quiescence by inducing low ERK/P38 signal ratio (2). Induced tumor dormancy by crosstalking with AXL and GAS6 (3). GAS6 can regulate tumor dormancy by being combined with TAM receptors such as AXL and Tyro3	The dormant tumor cells can express abundant TGF-β2 to maintain the dormant state. GAS6 can be derived from BMSCs or osteoblasts	Taichman et al., 2013; Yumoto et al., 2016; Goddard et al., 2018; Axelrod et al., 2019; Hen and Barkan, 2020
	Regulating	TGF-β3	Promoting the proliferation and metastasis of head and neck cancer by inducing matrix-specific protein periostin	TGF-β family members are involved in tumor cells proliferation, differentiation, and tumor dormancy.	Qin et al., 2016
	Promoting	atRA	Increasing the expression of TGF-β2 via activating p38 and p27 MAPK-dependent pathways	TGF-β2 mainly provokes dormancy during tumor progression	Linde et al., 2016
	Promoting	BMP4	Inducing breast cancer dormancy via activating SMAD1/5 signaling	BMP is one subgroup of the TGF-β family, which can influence the induction of tumor dormancy	Gao et al., 2012
	Promoting	BMP7	Inhibit tumor cell growth and drive CSCs into dormancy by mediating the expression of N-myc downstream-regulated gene 1 (NDRG1) and activating p38 MAPK and p21	BMP7 could induce dormancy of prostate cancer	Kobayashi et al., 2011; Widner et al., 2018
	Promoting	CXCL12	(1). Inducing BMSCs to migrate to cancer site (2). Triggering DTC dormancy by promoting the exchange of cell-cell information and cellular adhesion between MSCs and DTCs (3). Regulating tumor dormancy by mediating the tumor inflammatory responses	CXCL12 is a classic chemokine that can promote tumor cells in the bone marrow to enter dormancy.	Balkwill, 2004; Widner et al., 2018; Susek et al., 2018
	Inhibiting	IL-10	Inhibit the growth of lymphoma and leukemia cells by reducing the secretion of interleukin IL-10	MSCs can regulate the expression of IL-10 to induce tumor dormancy	Lee et al., 2019
	Regulating	NE	(1). Binding with β2-adrenergic receptors (2). Modulating the expression levels of GAS6	The neurons can regulate tumor dormancy through releasing NE	Widner et al., 2018
	Promoting	MiR-127 MiR-197 MiR-222 MiR-223 MiR-23b	(1). Driving breast cancer cells into quiescence through reducing the expression of CXCL12 (2). The DTCs can promote BMSCs to express abundant distinct miRNAs such as miR222/223 and miR23b, all of which can result in the dormancy of certain DTCs by suppressing the TGF-b pathway	Dormant breast cancers could promote MSC to release exosomes including distinct miRNA such as miR-127, -197, -222, and -223 The quiescent phenotype of tumor cells can be reversed by antagomiR-222/223	Guasch and Blanpain, 2004; Ono et al., 2014; Bliss et al., 2016
	Prompting	Cell cannibalism	The cannibalism of MSCs could also drive MDA-MB-231 BCCs to enter dormancy under demanding conditions	The cannibalized MDA-MB-231 BCCs obtain a similar cell phenotype with dormant tumor cells	Bartosh et al., 2016
	Promoting	TWIST1	Inducing tumor micrometastatic dormancy by activating tumor growth-inhibitory signals pathway	The expression of TWIST1 is upregulating after being co-cultured with BMSCs in 3D non-adherent culture platforms	Tran et al., 2011
	Promoting	LOX JNK p38	Driving MDA-MB-231 BCCs into dormancy through cooperating with TWIST1	TWIST1 can regulate micrometastatic dormancy by interacting with LOX, JNK, and p38	El-Haibi et al., 2012
	Promoting	SASP	(1). Activating cytokine and chemokine signaling (2). Inhibiting cell proliferation and vascular development (3). Initiating inflammatory/immune response	There is an obviously upregulating of the expression of CXCL1, CXCL2, GCSF IL-1α, IL-1β, IL-8, and PAI-1, all of which are integral to the expression of the senescence-associated secretory phenotype (SASP)	Özcan et al., 2015; Bartosh et al., 2017
	Promoting	Cellular morphology	Inducing tumor cell dormancy by regulating the changes of extracellular matrix such as hypoxia and ECM detachment	Cellular morphology changes show many analogous features with cell cannibalism	Sosa et al., 2013
	Promoting	JNK SAPK FTIs	(1). The breast tumor cell morphology change makes these cells enter into dormancy through activating the JNK/SAPK signaling pathway (2). FTIs can induce breast cancer cells into reversible dormancy by undergoing morphology	There are direct and indirect links between morphology changes and tumor dormancy.	Chatterjee and van Golen, 2011
hUCMSCs	Promoting	β-catenin c-Myc Wnt	(1). Driving lung cancer cells to be arrested in the G0/G1 phase (2). Driving hepatocellular cancer cells are arrested in the S phase (3). Downregulating the expression of β-catenin and c-Myc	The detailed mechanisms of hUCMSCs on tumor dormancy may include inducing cell cycle arrest, promoting tumor cell apoptosis, as well as inhibiting the migration of cancer cell	Yuan et al., 2018
TA-MSCs	Promoting	TRAIL CXCL12 TGF-β MMPs microRNAs	(1). Drive epithelial tumor cells to enter dormancy during the tissue remodeling stage (2). Inhibit angiogenesis by expressing inhibitory factors (3). Initiating a cell cannibalism behavior	TA-MSCs often play an important role in the progression of tumor growth and metastasis.	Lee et al., 2012; Li et al., 2019
AMSCs	Promoting	miRNAs Wnt TGF-β	(1). Regulating the tumor dormancy of breast cancer by secreting multiple circulating miRNAs (2). Arrested dormant BCCs in G0/G1 phase and S phase	The AMSCs are capable of transporting the exosomes carrying miRNAs to BCCs, which can target the Wnt and TGF-β signaling pathways, thus regulating tumor dormancy.	Mohd Ali et al., 2020

*MSCs, mesenchymal stem cells; BMSCs, bone marrow-derived mesenchymal stem cells; hUCMSCs, Human umbilical cord mesenchymal stem cells; TA-MSCs, tumor-associated mesenchymal stem cells; AMSCs, adipose tissue-derived mesenchymal stem cells; CSCs, cancer stem cells; DTC, disseminated tumor cells.*

### Bone Marrow-Derived Mesenchymal Stem Cells

In 1970, Friedenstein et al. (1970) reported the BMSCs which were derived from bone marrow for the first time. In the next few years, subsequent studies further proposed that BMSCs could also derive from other organs and tissues, such as teeth, adipose tissue, skin, and amniotic fluid (Mushahary et al., 2018; Mastrolia et al., 2019). In the present review, we mainly focus on the role of BMSCs from bone marrow on tumor dormancy. In addition to BMSCs, the bone marrow niche also includes osteoblasts, osteoclasts, as well as endothelial cells, all of which could regulate tumor dormancy (Fornetti et al., 2018; Widner et al., 2018). Thus, Hu et al. (2020) have regarded the bone marrow as a “bone-tumor microenvironment” including various immune cells and cytokines that directly mediate tumor progression. Noteworthy, it has been proposed that bone marrow is the most common tissue where dormant tumor cells re-enter the cell cycle, leading to metastatic disease recurrence (Widner et al., 2018). Pantel et al. (2009) further reported that bone marrow might serve as a common homing site for dormant tumor cells, and can increase the interaction between tumor cells and BMSCs. It has also been suggested that DTCs could crosstalk with BMSCs in the bone marrow (Bliss et al., 2016; Cammarota and Laukkanen, 2016), and there are abundant secreted factors such as all-trans retinoid acid and BMP-7 in the bone marrow microenvironment, which can inhibit the growth of DTCs and drive tumor cells into dormancy (Linde et al., 2016; Gay and Malanchi, 2017). The BMSCs mainly reside in the bone marrow stem cell niche, perivascular niche, and endosteal niche (Phan and Croucher, 2020). Therefore, BMSCs can interact with these niches and DTCs/CSCs directly or indirectly (Patel et al., 2014; Casson et al., 2018), including producing factors, secreting exosomes and extracellular vesicles and exosomes, cellular cannibalizing BMSCs, as well as inducing cellular morphology changes. All in all, once the DTCs enter into the bone marrow, the BMSCs will be recruited to interacting with these tumor cells (Walker et al., 2016).

#### Producing Factors

Current studies have reported that BMSCs can secrete a variety of microRNAs and various cell cytokines such as TGF-β family molecules, chemokine ligand 12 (CXCL12), growth arrest-specific protein 6 (Gas6), and so on (Decker et al., 2017; Talukdar et al., 2019; Hu et al., 2020). These substances can directly interact with target cells or be indirectly stored inside exosomes, leading to the dormancy of DTCs and CSCs. TGF-β family members are very common in various cells and organs, which not only directly affects inflammation response and tissue repair, but also regulates cell proliferation, differentiation, and tumor dormancy (Seoane and Gomis, 2017). The roles of TGF-β family members on tumor progression are different at distinct stages (Prunier et al., 2019). It has been reported that TGF-β family members could suppress tumor cell proliferation at an early stage but promote tumor metastasis at an advanced stage (Papageorgis, 2015), which may be caused by different subtypes of TGF-β can activate distinct signal pathways and produce diverse chemokines. For example, TGF-β1 has a dual role in tumor dormancy, promoting breast cancer cell proliferation by inhibiting the angiogenic dormancy (Ghajar et al., 2013; Jahanban-Esfahlan et al., 2019) while inducing tumor cell latency of squamous cell carcinoma (Brown et al., 2017). However, TGF-β2 mainly provokes dormancy during tumor progression, which can drive CSCs and DTCs into quiescence by inducing a low ERK/P38 signal ratio (Goddard et al., 2018; Hen and Barkan, 2020), and conversely, these dormant tumor cells can express abundant TGF-β2 to maintain the dormant state (Johnson et al., 2016; Yumoto et al., 2016). Notably, during this induced dormancy progress, TGFβ2 is required to crosstalk with AXL and GAS6 (Yumoto et al., 2016).

Growth arrest-specific protein 6 can be derived from BMSCs or osteoblasts and plays an important role in the progression of tumor cells dormancy and reactivation by being combined with TAM receptors such as AXL and Tyro3 (Taichman et al., 2013; Axelrod et al., 2019). Decker et al. (2017) have proposed that neurons could also govern tumor dormancy through releasing norepinephrine, which can bind with β2-adrenergic receptors and modulate the expression levels of GAS6, thus promoting or inhibiting cancer cell proliferation (Widner et al., 2018). Moreover, it has been reported that all-trans retinoid acid could increase the expression of TGF-β2 by activating p38 and p27 MAPK-dependent pathways (Linde et al., 2016). In contrast, there are few reports about the influence of TGF-β3 on tumor dormancy, which proposed that TGF-β3 could promote the proliferation and metastasis of head and neck cancer by inducing matrix-specific protein periostin (Qin et al., 2016).

Bone morphogenetic proteins, one subgroup of the TGF-β family, also influence the induction of tumor dormancy. Especially, BMP4 and BMP7 are two common objects of dormancy study with mouse models, in which it has been discovered that BMP4 could induce dormancy of breast cancer (Gao et al., 2012) and BMP7 could induce dormancy of prostate cancer (Kobayashi et al., 2011). Furthermore, BMP4 was capable of inducing tumor dormancy via activating SMAD1/5 signaling (Gao et al., 2012) and BMP7 can inhibit tumor cell growth and drive CSCs into dormancy by mediating the expression of N-myc downstream-regulated gene 1 and activating p38 MAPK and p21 signaling pathways (Kobayashi et al., 2011; Widner et al., 2018). It has also been suggested that TGFβ and BMP both can stimulate MSCs to differentiate into osteoblasts, which were involved in the formation of the bone-tumor microenvironment (Chen et al., 2012).

In addition to BMSCs, other bone marrow stromal cells such as endothelial cells and osteoblasts can also secrete CXCL12, acting as an inducer for BMSCs to migrate to cancer sites (Widner et al., 2018). CXCL12 is capable of binding with the C-X-C motif chemokine receptor 4 (CXCR4) expressed on the surface of DTCs, directly triggering DTC dormancy through promoting the exchange of cell-cell information and cellular adhesion between BMSCs and DTCs (Balkwill, 2004; Widner et al., 2018). In addition, the CXCR4/CXCL12 axis has been reckoned as an important marker and mediator of tumor cells homing to bone (Reagan and Rosen, 2016). The bone marrow was capable of secreting a high level of CXCL12, which could be regulated by TGF-β family members (Yu et al., 2017) and microRNAs (Dittmer, 2017). For instance, connexin 43-based gap junction, a channel connected between breast cancer and BMSCs, can be used to exchange microRNAs, thus suppressing the synthesis of CXCL12 (Lim et al., 2011). Further studies have discovered that hematopoietic stem cells (HSCs) can also express CXCR4 (Sugiyama et al., 2006). Therefore, HSCs can be home to bone marrow and remain dormant with the combination of CXCL12 and CXCR4 (Hoggatt et al., 2016). On the other hand, CXCL12 also mediates the tumor inflammatory responses by interacting with various immune cells, which can either promote or suppress tumor dormancy (Susek et al., 2018).

#### Producing Exosomes and Extracellular Vesicles

Extracellular vesicles (EVs) are defined as cell structures composed of proteins, miRNAs, nucleic acid, and biological signaling molecules (vakhshiteh et al., 2019). Exosomes are one subtype of EVs with diameters between 20 and 100 nm (Susek et al., 2018). EVs can be derived from various subcellular compartments, and each type of exosome or EVs includes specific molecular constituents and plays distinct roles in tumor development (El Andaloussi et al., 2013; Pitt et al., 2016). For example, the fibroblast-secreted exosomes are involved in the migration of breast tumor cells via regulating autocrine Wnt–PCP (planar cell polarity) signaling pathway (Luga et al., 2012). In addition, the cancer cell-derived exosomes can promote tumor formation and metastasis by regulating the functions of surrounding noncancerous cells (Peinado et al., 2012), promoting angiogenesis, and neutrophil infiltration (Ono et al., 2014). Furthermore, the exosomes derived from stromal cells are capable of transferring their contents into tumor cells, thus influencing the progression of cancer (Luga et al., 2012; Roccaro et al., 2013). The emphasis in this paper is the effects of BMSC-derived exosomes and EVs on tumor dormancy. The BMSC-derived EV cargo is composed of tumor-supportive molecules and various miRNAs such as miR-205, miR-31, miR23b, and miR21, all of which are key mediators in tumor dormancy (Vallabhaneni et al., 2017; Casson et al., 2018).

Firstly, it has been demonstrated that BMSCs can directly crosstalk with tumor cells via these BMSC-derived EVs (Ono et al., 2014). In other words, the exosomes and EVs can be utilized to transfer proteins and RNAs from BMSCs to tumor cells. BMSC-derived exosomes and EVs are like a two-edged sword, which can either promote or inhibit tumor progression (vakhshiteh et al., 2019; Zhao et al., 2020). According to the latest discoveries, tumor-derived EVs are capable of promoting tumor cell proliferation (Cappariello and Rucci, 2019). However, the BMSC-derived EVs can transfer dormancy initiating factors such as miRNAs into tumor cells (Widner et al., 2018), thus leading to the formation of tumor dormancy.

Although components of exosomes and EVs are multitudinous, the most commonly used in tumor dormancy researches is miRNAs, which can regulate tumor dormancy through modulating the expression of CXCL12 and the duration of TGF-β signaling. For example, current studies have discovered that dormant breast cancers could promote MSC to release exosomes including distinct miRNA such as miR-127, -197, -222, and -223, driving breast cancer cells into quiescence through reducing the expression of CXCL1 (Hanahan and Folkman, 1996; Bliss et al., 2016). Besides, the DTCs and MSCs can easily crosstalk within bone marrow as their anatomical locations are near, and the DTCs can promote BMSCs to express abundant distinct miRNAs such as miR222/223 and miR23b (Ono et al., 2014), and conversely, these miRNAs can result in the dormancy of certain DTCs by suppressing the TGF-b pathway (Guasch and Blanpain, 2004). On the other hand, it has been suggested that the quiescent phenotype can be reversed by antagomiR-222/223 (Bliss et al., 2016). More importantly, another study has proposed that the miR222/223 can also result in tumor cell drug resistance (Hanahan and Folkman, 1996), and there are abundant miR222/223 in the BMSC-derived exosomes and EVs, indicating that BMSCs can induce drug resistance of cancer (Ruksha, 2019). Based on these discoveries, antagomir-222/223 can be used to target dormant breast cancer cells, which may be a promising therapeutic strategy.

There are many interactions between cancer cells and BMSCs in the bone marrow via paracrine secretion or gap junctional intercellular communication, which can also be used to transport miRNAs. For instance, it has been suggested that miRNAs can be exchanged between BMSCs and BBCs through gap junctional intercellular communication established by bone marrow stroma^48^, which directly controls the proliferation of breast cancer cells. In addition to BMSC-derived exosomes and EVs, many other circulating exosomes and EVs such as OS-derived, cancer-derived, and bone-derived, all of which can also be used as a promising clinical tool for clinical management and monitoring, as well as an index to screen therapeutic efficiency (Cappariello and Rucci, 2019).

#### Cannibalizing Bone Marrow-Derived Mesenchymal Stem Cells

Cell cannibalism can occur among the homotypic type of cells or heterotypic types of cells (He et al., 2013). Cell cannibalism is also thought to be a live-cell feeding behavior, which is not just about obtaining resources from other cells and is distinct from traditional macrophages phagocytosis, a live-cell entosis, as well as cytoplasm emperipolesis (Overholtzer et al., 2007). Bartosh et al. have discovered that MDA-MB-231 breast cancer cells (BCCs) could cannibalize BMSCs in 3D co-cultures, and the cannibalism of MSCs could also drive MDA-MB-231 BCCs to enter dormancy under demanding conditions (Bartosh et al., 2016). During the cannibalism of this progress, BMSCs quickly encapsulate clusters of MDA-MB-231 BCCs and change their phenotype of tumor cells, and then BMSCs are internalized (Bartosh et al., 2016). Further studies have indicated that the cannibalized MDA-MB-231 BCCs obtain a similar cell phenotype with dormant tumor cells (Bartosh et al., 2016). At the same time, BMSCs could also drive MDA-MB-231 BCCs into quiescence/dormancy through producing factors and exosomes. In addition to secreting various factors such as microRNA and signal proteins, a significant increase of expression of transcription factor TWIST1 after being co-cultured with BMSCs in 3D non-adherent culture platforms has also been observed, which can activate tumor growth-inhibitory signals pathway, and thus leading to the micrometastatic dormancy (Tran et al., 2011; Bartosh et al., 2016). Besides, the expression of lysyl oxidase and Jun N-terminal kinase also increased in these tumor cells, both of which can cooperate with TWIST1 to drive MDA-MB-231 BCCs into dormancy (El-Haibi et al., 2012). In other words, TWIST1 can regulate micrometastatic dormancy by interacting with lysyl oxidase, Jun N-terminal kinase, and p38. According to MDA-MB-231BCCs phenotype analysis, there is an obvious up-regulation of various cytokines/chemokines such as CXCL1, CXCL2, GCSF IL-1α, IL-1β, IL-8, and PAI-1 (SERPINE1), all of which are integral to the expression of the senescence-associated secretory phenotype, an important mediator in regulating tumor dormancy and relapse, which could induce tumor dormancy via activating cytokine and chemokine signaling, inhibiting cell proliferation and vascular development and initiating inflammatory/immune response (Özcan et al., 2015; Bartosh, 2017).

Cell cannibalism and dormancy represent cell survival status, in which cell growth might arrest or slow down. But there is no doubt that the cannibalism of BMSCs directly associates with tumor dormancy in tumor niches. However, the characteristics of cannibalistic tumor cells are distinct from traditional CSCs, indicating that these dormant cells may be a new cell population (Mitra et al., 2015). Further studies have proposed that the cannibalistic BCCs can recover the proliferation ability once they return to suitable surroundings (Bartosh et al., 2016). Moreover, one study has suggested that BMSCs with different sources might show distinguish cannibalism effects, and the detailed cues are still unclear (Castellone et al., 2013). Likewise, our observed results are linked to cell cannibalism, but the pathophysiological effect is not completely understood. Cell cannibalism, which has been proposed for many years was initially used to assess the deterioration of cancer (Gupta and Dey, 2003). Furthermore, current studies have also found that cell cannibalism could promote tumor formation and tumor gene transfer (Gupta and Dey, 2003; Bartosh et al., 2016). In conclusion, cell cannibalism may be just one of the ways of cellular interaction. With the development of the co-culture model, more information about the properties of BMSCs and cell cannibalism will be discovered, contributing to new methods of cancer treatment.

#### Inducing Cellular Morphology Changes

Compared with cell cannibalism, cellular morphology change is a self-degradative process occurring inside the cell to avoid apoptosis through degrading organelles such as mitochondria (Levine and Kroemer, 2008). Cellular morphology changes show many analogous features with cell cannibalism (Walker et al., 2016). Some studies have proposed that morphology could result in cell dormancy, which is regulated by extracellular matrix (Kai et al., 2019). For example, when being in microenvironmental stresses such as hypoxia and extracellular matrix detachment, tumor cells are capable of quickly entering into a dormancy-like state (Sosa et al., 2013). These dormant tumor cells usually reside in secondary sites, resulting in tumor recurrence after a long time. For instance, Chatterjee et al. discovered that breast tumor cell morphology changes make these cells enter into dormancy by activating the JNK/SAPK signaling pathway. Besides, farnesyl transferase inhibitors can induce breast cancer cells into reversible dormancy by undergoing morphology, which has also been used in clinical anti-tumor research (Chaterjee and van Golen, 2011).

Further study proposes that autophagy is involved in the formation of the “stemness” of tumor cells (Talukdar et al., 2018), which also explains why normal tumor cells can show similar characteristics with CSCs. In conclusion, these studies suggest there are direct and indirect links between morphology changes and tumor dormancy. However, whether MSCs can induce tumor dormancy through cellular morphology changes is still unclear, and the study on the relationship between MSCs and morphology changes is rare. To better understand the mechanisms of tumor dormancy, how MSCs regulate morphology should be investigated in future studies.

### Human Umbilical Cord Mesenchymal Stem Cells

Human umbilical cord mesenchymal stem cells (hUCMSCs), a type of adult stem cell stemming from the umbilical cord matrix, have been discovered to possess the ability to inhibit tumor cell proliferation and metastasis, and are regarded as a significant potential treatment tool for solid tumors (Ciavarella et al., 2011; Yuan et al., 2018). hUCMSCs possess the general characteristics of MSCs, such as self-renewal and multi-directional differentiation ability, and have stronger expansion capability and lower risk of virus contamination during the process of cell-based therapy than BMSCs. Subramanian et al. have discovered that hUCMSCs do not differentiate into tumor-associated fibroblasts during the interaction with the tumor microenvironment, indicating that hUCMSCs are safer than BMSCs (Subramanian et al., 2012).

To date, plenty of studies have focused on the cross-talk interaction between hUCMSCs and tumor cells, and it has been shown that hUCMSCs could either promote or inhibit tumor development (Yuan et al., 2018). For instance, Shen et al. have revealed that IFNβ gene-transfected hUCMSCs could significantly suppress the growth of human triple negative breast carcinoma cell lines MDA-MB-231 and Hs578T (Shen et al., 2016). It has also been reported that hUCMSCs could attenuate proliferation and induce apoptosis of glioma cells through regulating cell cycle progression, downregulating the expression of anti-apoptotic genes, β – catenin, c-Myc, as well as upregulating the level of apoptotic genes such as caspase-3 and caspase-9 (Yang et al., 2014; Yuan et al., 2018). By contrast, Yang et al. have discovered that hUCMSCs could promote tumor proliferation and metastasis, which have been observed in a lymph node, carcinoma, gastric cancer, and esophageal carcinoma (Yuan et al., 2018). To sum up, the effects of hUCMSCs on tumors are different, depending on the types of cancer. In the following part, the relationship between hUCMSCs and tumor dormancy is discussed.

In terms of tumor dormancy, one study has shown that hUCMSCs could support tumor dormancy via induction of cell cycle arrest in specific phases, leading to microscopic tumor cell clusters enter a balance between proliferation and apoptosis (Yuan et al., 2018). However, different types of tumor cells might be arrested in distinct cell cycle phases. For example, hUCMSCs can drive lung cancer cells to be arrested in the G0/G1 phase, while hepatocellular cancer cells are arrested in the S phase (Yuan et al., 2018). In addition to hUCMSCs, Lee et al. have discovered that BMSCs could also suppress the proliferation of hematologic malignancy by inducing tumor cell cycle arrest (Lee et al., 2019).

In addition, another study has indicated that hUCMSCs could not only inhibit the proliferation of human lung cancer cells and human hepatocellular carcinoma cells but also induce these tumor cells into dormancy by downregulating the expression of β-catenin and c-Myc, two key players in the Wnt signaling pathway (Yuan et al., 2018). In conclusion, these studies suggest that the detailed mechanisms of hUCMSCs on tumor dormancy may include inducing cell cycle arrest, promoting tumor cell apoptosis, as well as inhibiting the migration of cancer cells. Moreover, for a better understanding of the effect of hUCMSCs on tumor dormancy, the expression of dormancy biomarkers has been further analyzed. It is found that the level of ephrin receptor, a common tumor dormancy marker, obviously increases, indicating that tumor cells might have entered dormancy (Yuan et al., 2018). There is still a long way in studying the effects of hUCMSCs on tumor dormancy, and studies have shown that the specific mechanism of hUCMSCs on tumor dormancy is different in terms of the types of tumor cells.

### Tumor-Associated Mesenchymal Stem Cells

Tumor-associated mesenchymal stem cells, derived from normal MSCs in the tumor microenvironment, are distinct from other organ-derived MSCs such as BMSCs and AMSCs (Trivanovic et al., 2016). TA-MSCs, which actively regulate tumor growth and metastasis during cancer progression, have been discovered in various types of tumors such as gastric, liver, breast, prostatic, and ovarian cancer (Shi et al., 2017). Besides, it has been found that TA-MSCs influence tumor progression through secreting cytokines and chemokines (Shi et al., 2017). These factors are contributing to tumor metastasis and drug resistance. Likewise, TA-MSCs can be differentiated into myofibroblasts and support the survival of CSCs and angiogenesis. All in all, TA-MSCs often play an important role in the progression of tumor growth and metastasis. However, it has been observed that TA-MSCs could also suppress the proliferation of epithelial tumor cells and drive them to enter dormancy during the tissue remodeling stage (Li et al., 2019). Next studies have found that TA-MSCs could not only inhibit angiogenesis by expressing inhibitory factors such as TNF-related apoptosis-inducing ligand, CXCL12, TGFβ, matrix metalloproteinases, and microRNAs but also initiate a cell cannibalism behavior (Lee et al., 2012; Li et al., 2019). The above effects are both involved in the regulation of tumor dormancy. At present, there is no clear definition and classification between MSCs and TA-MSCs, which, in most studies are discussed together. Earlier in this review, how MSCs influence tumor dormancy is analyzed and these mechanisms could also be discovered during the process of interaction between TA-MSCs and the tumor microenvironment.

### Adipose Tissue-Derived Mesenchymal Stem Cells

In recent years, AMSCs have been seen as one of the most promising anticancer treatments in cancer-targeted therapy (Ramdasi et al., 2015). For example, it has been proposed that the AMSCs can regulate the tumor dormancy of breast cancer via secreting multiple circulating miRNAs (Mohd Ali et al., 2020) with the stromal AMSCs and BCCs being co-cultured in a non-contact microenvironment model. After 48h of co-culture, the proliferation of BCCs is significantly inhibited, which may be due to these tumor cells are driven to the dormant state by the AMSCs. Further studies have proposed that this inhibitory effect is correlated with the distribution of the cell cycle and most dormant BCCs were arrested in G0/G1 phase and S phase. In contrast, when returning to the original culture condition, these co-cultured dormant cells can reenter cell regulation. To be more specific, the AMSCs are capable of transporting the exosomes carrying miRNAs to BCCs, which can target the Wnt and TGF-β signaling pathways, thus regulating tumor dormancy.

## Clinical Application Potentials

Stem cell-based therapies have been used in anticancer researches for many years and MSCs are considered the most potent therapy tools. The reasons are summarized below. Firstly, MSCs can directly restrain the growth of some tumors by inhibiting the vasculature or arresting the cell cycle. Secondly, the engineered or modified MSCs have also been applied as targeted anticancer carriers for gene therapy (Li et al., 2020), which are more efficient and safer than naive MSCs. Moreover, MSCs can cross-talk with CSCs/DTCs via paracrine mechanisms, and regulate the biological activity of tumors (Lin et al., 2019), for example, AMSCs could suppress the growth of lung carcinoma cells by secreting cytokines such as interferon (IFN)-β and tumor necrosis factor-related apoptosis-inducing ligand (Cortes-Dericks and Galetta, 2019). In this review, the influence of MSCs on tumor dormancy is summarized and the ways of MSCs inducing CSCs and DTCs into dormancy is shown, helping to develop more promising treatment therapies.

During the past decade, researchers have realized that dormant tumor cells might be the primary reasons for therapy failure as the drug-resistant cells and dormant cancer cells share many similar biological features such as heterogeneity and plasticity (Luga et al., 2012), According to current clinical studies, there are two alternative strategies based on tumor dormancy. One way is to reawaken dormant tumor cells before treatment to make these cells susceptible to the therapies such as chemotherapy, immunotherapy, targeted therapy, and radiotherapy. For instance, one study has suggested that DTCs can be eliminated by manipulating their surrounding microenvironment to influence their communication with tumor stromal cells (Peinado et al., 2012). The other way is to drive dormant tumor cells into a perpetual dormant state. Because disseminated cancer cells could enter into the perivascular niche and remain dormant for decades, previous therapies can be applied to prolong the dormancy periods to prevent tumor metastasis and recurrence. For example, one clinical study has discovered the survival of patients with glioblastoma multiforme was significantly reduced, which may be caused by losing the control of dormant tumor cells. However, maintaining a perpetual tumor dormant state or preventing tumor cells from establishing dormancy can prolong the life of these patients (Ono et al., 2014).

More specifically, with the rapid development of stem cell-based therapies and molecular biology, great progress has been achieved in MSCs-based antitumor studies. It has shown that MSCs can be used to regulate tumor dormancy, which will provide more effective clinical strategies to prevent or delay cancer recurrence. Based on tumor dormancy, the potential clinical applications of MSCs on anticancer studies are summarized below.

Firstly, the MSCs and their exosomes can be used as carriers to target cancers. It is acknowledged that MSCs are capable of homing to sites of tissue injury and tumor microenvironment by reducing immune-inflammatory responses (Eltoukhy et al., 2018b). Based on this feature, researchers have proposed that MSCs could be used as carriers to deliver nanoparticles, anti-tumor drugs, proteins, lipids, DNAs, mRNAs and miRNA (Eltoukhy et al., 2018b; Kwon et al., 2019). For example, one study reported that internalization of paclitaxel-loaded nanoparticles to MSCs can achieve promising antitumor efficacy, which is caused by sustained release of the encapsulated paclitaxel to the tumor microenvironment (Wang et al., 2018). Another study proposed that BM-MSCs can induce breast CSCs into dormancy through releasing inhibitory miRNA such as antagomiR-222/223 (Ono et al., 2014; Eltoukhy et al., 2018b). Moreover, Lai et al. have conducted more detailed studies and discovered that the exosomes derived from MSCs are better candidates for drug delivery due to natural features such as easy isolation, strong tolerability, as well as the ability to bind with the plasma membranes (Vallabhaneni et al., 2017). It is believed that MSCs–derived exosomes can be used to prevent the recurrence of cancer. Although in the experimental investigation stage, these studies have developed a new therapeutic strategy for cancer.

Secondly, cannibalism of MSCs can be used to assess the prognosis of cancer. Current studies have suggested that the cannibalism of MSCs can promote tumor dormancy, and happen in highly aggressive tumors most frequently (Bartosh et al., 2016). Therefore, Thomas et al. have proposed that there is a logical cause-and-effect relationship between cell cannibalism and dormancy (Bartosh et al., 2016). To be more specific, cannibalistic cells can be used to identify cancer phenotype, which will provide new windows for cancer therapeutic intervention and prognostic evaluation. Furthermore, with the improvement of the 3D coculture model, more mechanisms underlying the mediation of cell cannibalism by MSCs will be unraveled and more antitumor tools can be used in clinical application.

Third, the formation of tumor dormancy can be intervened by targeting the key players of interaction between MSCs and tumor cells. Until now, with the discovery of more and more key players regulating organ-specific metastasis and dormancy (Neophytou et al., 2019), targeted therapy has been frequently used in clinical applications. Directly targeting tumor cells is highly effective cancer therapy. However, the MSCs can interact with CSCs and drive them into a state of cycling dormancy through secreting proteins, exosomes, and cytokines, which will make these CSCs resist targeted therapy (Lim et al., 2011; Patel et al., 2012; Ono et al., 2014). Therefore, to make these CSCs drug-sensitive, the interaction between the CSCs and MSCs needs to be disrupted (Eltoukhy et al., 2018a). One of the most promising strategies for reducing bone metastasis is to target dormancy-associated niches. In clinical practice, bone metastasis is reckoned as an indicator of poor prognosis in most cases. With the description of the “bone metastatic niche” concept, which consists of connective tissues, bone stromal cells, and signaling molecules, some researchers have proposed that bone metastatic niche can be targeted to prevent or delay the progression of bone metastasis (Ren et al., 2015). For example, BMSCs, one subtype of bone stromal cells, can either promote or impede the progression of tumor dormancy by secreting BMP7, TGFβ2, GAS6 (Kaplan et al., 2006; Decker et al., 2017; Hu et al., 2020; Li et al., 2020). Targeting the bone metastatic niche can disrupt the interaction between MSCs and tumor cells, thus restraining the formation of tumor dormancy. Consistent with these discoveries, another study has suggested that dormant tumor cells could become sensitive to normal cytotoxic therapies by targeting at DTC dormant niche (Saito et al., 2010). All in all, directly targeting the mechanisms underlying tumor dormancy is a new clinical cancer strategy.

Finally, some researchers have proposed that immune therapy combined with dormancy therapy would develop a new cancer therapy (Linde et al., 2016). For example, immunotherapy has been successfully used in maintaining the dormancy of HIV, which has many similarities to tumor cells (Boire et al., 2019). Simultaneously, MSCs can either suppress or enhance the immune function of cancer cells as influencing tumor proliferation (Roccaro et al., 2013). In conclusion, a better understanding of the immunobiological pathways of dormant tumor cells will help study the mechanisms of how MSCs mediate tumor dormancy (Kleffel and Schatton, 2013).

## Challenges and Prospects

Tumor dormancy has attracted more and more attention from researchers. However, related investigations are limited by the lack of classical models that can represent the human condition. The essential effects of MSCs on tumor dormancy have been discussed. The influence of bone marrow microenvironment such as fibroblasts, endothelial cells, and immune cells can also not be ignored (Reddy et al., 2012). However, it is difficult to recreate the structure of the bone marrow, limiting the studies of other cells within the bone marrow. In the past several years, many researchers have used the 2D model of the bone marrow to replicate the interaction between cellular and soluble factors (Chong Seow Khoon, 2015). However, as time goes on, traditional 2D co-cultures platforms have shown deficiencies (Widner et al., 2018). For instance, 2D co-cultures could not adequately mirror the natural bone marrow microenvironment and physiological conditions of cells, which greatly limits the therapeutic testing and system modeling *in vitro* (Bartosh et al., 2016).

To better study the interactions between bone marrow and tumor dormancy, it is essential to model a platform microenvironment where different types of cells can be co-cultured and cell phenotype can be analyzed (Eltoukhy et al., 2018b). Fortunately, in recent years, with the emergence of 3D bioprinting and engineered organotypic models, an increasing number of fundamental problems of tumor biology have been addressed. For example, an engineered organotypic microvascular niche was utilized to study the interaction between endothelial cells and breast cancer cells. The results showed that endothelial-derived thrombospondin-1 could directly induce sustained breast cancer cells quiescence (Ghajar et al., 2013). Similarly, a 3D system can be created to recapitulate the bone microenvironment (Gungor-Ozkerim et al., 2018). This system can be used to better understand the biological properties of MSCs and mirror natural conditions *in vivo* (Clark et al., 2018). Besides, the model can be used to create an MSCs-tumor stem cell co-culture system, which greatly helps us investigate the interaction between the tumor cells and their niches (Sart et al., 2014; Moore et al., 2018). Likewise, we will easily select cell types and figure out which cells and niches support tumor dormancy (Eltoukhy et al., 2018b). However, though these models have made a great contribution, the whole picture of dormancy-related cellular processes is still far from clear (Cole, 2009). Undoubtedly, only when the data collected from different dormancy models are analyzed together, may the stealth mechanisms of tumor dormancy start to be clarified.

In recent years, a plenty of mouse models have been established to reveal the mechanisms of various diseases. However, it is still difficult to validate the molecular mechanisms of dormancy in mouse models. First of all, due to the fact that tumor dormancy is a phenomenon that occurs at low frequency, developing a well-established mouse model of tumor dormancy is probably impractical. In addition, each mechanism of tumor dormancy has different complex characteristics, and current mouse models cannot precisely monitor and manipulate single or small clusters of tumor cells. On the other hand, even there are applicable conceptual models of dormancy, the result of these experimental studies is still difficult to be validated in patients. Therefore, it is very important to establish a sophisticated experimental dormant model and to isolate CSCs and DTCs from the tumor microenvironment, and then simulate the interaction between dormant tumor cells and the surrounding niches. Currently, since the dormant tumor cells are undetectable via traditional whole-body imaging tools (Linde et al., 2016), it is better to develop new diagnostic tools that can detect dormant tumor cells in patients as soon as possible. In the long run, if more biomarkers of dormant tumor cells can be identified before tumorigenesis or more quantitative methods and calculable tools can be used in clinical diagnosis, the recurrence and metastasis of cancer will be efficiently prevented.

## Conclusion

Whether MSCs promote or suppress tumor dormancy is still controversial. From an individual point of view, based on their sources, MSCs can be divided into two types: tumor-derived MSCs and other sources-derived MSCs. The tumor-derived MSCs are progeny of tumor cells, which mainly play a pro-tumor role during the tumor progression but other sources-derived MSCs play an anti-tumor role. However, this rule is not absolute and depends on the types of tumors. Aparts from being influenced by the sources of MSCs and the types of cancer, the effects of MSCs on tumor progression can also be influenced by the experimental models. Therefore, the interpretation of some experimental results was somewhat inconsistent, even the opposite. In the future, with the development of the 3D bioprinting technique, the modeling methods will be greatly improved.

In recent years, MSC-based therapies have been frequently used by clinical researchers. During these processes, MSCs are modified through the transfection of therapy genes and transportation of target agents, but the inherent characteristics and biological properties of MSCs are not changed. Combining with the studies of tumor dormancy, researchers can develop more modified MSCs to intervene in the progression of tumor dormancy. Although more detailed mechanisms underlying the interaction between MSCs and tumor cells needs further study, current studies are trying to apply engineered MSCs as treatment carriers to regulate tumor dormancy to prevent or delay tumor progression. All in all, the treatment potential of MSCs on tumors is infinite. Although it is difficult to apply these strategies clinically due to the limitations of current technical merit, there is no doubt that a huge step forward has been taken in the fight against cancer.

## Author Contributions

LZ and KZ drafted the review. HH generated the graphs. YY guided the construction of the manuscript. WL edited the review. JL and TL provided input on the scope and content of the review. All authors contributed to the article and approved the submitted version.

## Conflict of Interest

The authors declare that the research was conducted in the absence of any commercial or financial relationships that could be construed as a potential conflict of interest.

## Publisher’s Note

All claims expressed in this article are solely those of the authors and do not necessarily represent those of their affiliated organizations, or those of the publisher, the editors and the reviewers. Any product that may be evaluated in this article, or claim that may be made by its manufacturer, is not guaranteed or endorsed by the publisher.

## Abbreviations

CSCs: cancer stem cells
DTCs: disseminated tumor cells
MSCs: mesenchymal stem cells
HSCs: hematopoietic stem cells
DCCs: dormancy-competent cancer stem cells
DICs: dormancy-incompetent cancer stem cells
DTCs: Disseminated tumor cells
CTCs: circulating tumor cells
BMSCs: bone marrow-derived mesenchymal stem cells
AMSCs: adipose tissue-derived mesenchymal stem cells
TA-MSCs: tumor-associated mesenchymal stem cells
MAPK: Mitogen-activated protein kinase
TGF- β: transforming growth factor- β
BMPs: bone morphogenetic proteins
hUCMSCs: human umbilical cord-derived MSCs
CXCL12: chemokine ligand 12
Gas6: growth arrest-specific protein 6
CXCR4: C-X -C motif chemokine receptor 4
HSCs: hematopoietic stem cells
EVs: Extracellular vesicles
BCCs: breast cancer cells.
